# Sulfasalazine prevents lung injury due to intra-abdominal sepsis in rats: possible role of Nrf2 and angiopoietin-2

**DOI:** 10.1590/1414-431X2023e12698

**Published:** 2023-05-29

**Authors:** C. Dibekoğlu, Y. Uyanıkgil, O. Erbaş

**Affiliations:** 1 Department of General Surgery, Demiroğlu Bilim University, Istanbul, Turkey; 2 Department of Histology and Embryology, Ege University, Faculty of Medicine, Izmir, Turkey; 3 Department of Physiology, Faculty of Medicine, Demiroğlu Bilim University, Istanbul, Turkey

**Keywords:** Sulfasalazine, Sepsis, Sepsis induced-ARDS, Angiopoietin-2, Nrf2, Rats

## Abstract

This study aimed to investigate the effect of sulfasalazine in preventing and treating intra-abdominal sepsis-induced acute respiratory distress syndrome (ARDS) in a rat model. Forty male Wistar albino rats were used. The rats were randomly divided into four equal groups, and sepsis was induced in 30 rats by intraperitoneal administration of a fecal saline solution prepared from rat feces. Group 1: normal control (n=10) [non-surgical], Group 2: fecal intraperitoneal injection (FIP) (n=10) [untreated septic group], Group 3: FIP+saline (placebo) (n=10) [saline administered intraperitoneally], Group 4 (n=10): FIP+sulfasalazine [250 mg/kg per day administered intraperitoneally]. Computed tomography was performed and blood samples were collected for biochemical and blood gas analysis. The lungs were removed for histopathological studies. Statistically significant reductions in interleukin (IL)-6, IL1-β, tumor necrosis factor (TNF)-α, malondialdehyde (MDA), and angiopoietin-2 (ANG-2) levels were observed in the sulfasalazine group compared to the FIP+saline group (P<0.001). Nrf2 levels were significantly higher in the sulfasalazine-treated group than in the FIP and FIP+saline groups (P<0.01). Lung tissue scores were significantly reduced in the sulfasalazine group compared to the other sepsis groups. The Hounsfield unit (HU) value was significantly lower in the sulfasalazine group than in the FIP+saline group (P<0.001). PaO_2_ values were significantly higher in the sulfasalazine-treated group than in the FIP+saline-treated group (P<0.05). Sulfasalazine was shown to be effective in preventing and treating ARDS.

## Introduction

Sepsis is the leading cause of death in hospitalized patients. Although bacterial infections are usually the main cause of sepsis, it can also be caused by viral and fungal infections. Sepsis is not a disease but rather a combination of symptoms and signs (1). There is no specific treatment, and it is primarily symptomatic. Approaches that support organ function are an essential part of therapy (oxygen, intravenous fluids, antibiotics, and inotropic agents) (2). Acute respiratory distress syndrome (ARDS) is a clinical phenomenon that can be fatal due to severe sepsis. Severe sepsis is the most common etiologic cause of ARDS (3) and is associated with the highest mortality rate (4). Although the pathophysiology of ARDS is not fully understood, numerous important reports indicate that oxidative stress and inflammation are the major causes (5).

ARDS is a condition of respiratory failure with pulmonary edema of non-cardiac origin. ARDS is also commonly caused by sepsis from non-pulmonary causes, pneumonia, severe chest trauma, aspiration of gastric contents, inhalation of smoke or toxic gases, and less commonly pancreatitis, blood transfusion, and drug reactions (6). When the lungs are damaged by infection, trauma, or inflammation, inflammatory pathways are activated. As a result, proteinaceous fluid accumulates in the alveoli, making breathing difficult and disrupting gas exchange.

Computed tomography (CT) is the most valuable imaging modality for screening sources of infection, particularly for assessing pulmonary and intraperitoneal involvement in a patient with sepsis, and the most commonly used method for assessing pulmonary inflammatory changes and lung injury in ARDS (7). The use of pulmonary changes as quantitative scores to detect early-stage ARDS is a predictor of prognosis and disease survival using a CT-based scoring system (8).

Pro-inflammatory cytokines play a critical and unique role in the development of ARDS. As a result of epithelial cell detachment in the acute phase of ARDS, protein-rich hyaline membranes form on the damaged basement membrane. Neutrophils penetrate the damaged endothelium and fill the interstitium and then the alveoli with protein-rich edema fluid. In the alveoli, macrophages secrete cytokines, interleukin (IL)1-β, 6, 8, 10, and tumor necrosis factor-α (TNF-α), which act locally to activate neutrophils and initiate chemotaxis. Activated macrophages additionally secrete angiopoietin-2 (ANG-2) and 1 (ANG-1) and reactive oxygen species (ROS). ANG-2 is a pro-inflammatory agent that causes deterioration of barrier function, provides epithelial and endothelial apoptosis, and is readily detected in plasma (9). IL-1 stimulates the production of extracellular matrix by fibroblasts. In addition, neutrophils can release other pro-inflammatory molecules such as oxidants, proteases, leukotrienes, and platelet-activating factors (PAF) in the alveoli (10).

Endothelial permeability, one of the critical pathophysiological features of ARDS, increases because of oxidative stress. Nuclear factor erythroid 2-associated factor 2 (Nrf2) is a transcription factor and regulates the expression of antioxidant proteins that protect against oxidative damage (11).

Histopathologically, diffuse alveolar damage (DAD) manifests with neutrophilic infiltration, alveolar hemorrhage, and hyaline membrane formation (12). After the first 24 h, the lungs appear morphologically heavy, congested, and often dark red-blue due to congestion. The main histological features in this exudative phase are the formation of hyaline membranes, edema, and acute interstitial inflammation (13).

Sulfasalazine is an old and safe antirheumatic drug used to treat various inflammatory diseases such as rheumatoid arthritis and ulcerative colitis (14). However, its mechanism of action remains unclear. As described by Smedegård et al. (15), the effect of sulfasalazine in rheumatoid arthritis is probably mediated by various immunomodulatory and anti-inflammatory effects. Despite many years of basic and clinical research, there is still no effective pharmacotherapy for this syndrome. Treatment remains primarily supportive with a conservative fluid management strategy and lung-sparing ventilation. Therefore, it is critical to explore the pathogenesis and pathophysiology of ARDS to identify novel targeted therapies for this condition (16).

Given the previous information, we designed this study to investigate the effects of sulfasalazine in intra-abdominal sepsis-related ARDS. In addition to its known anti-inflammatory effects, we hypothesized that sulfasalazine exerts these effects via the Nrf2 and ANG-2 signaling pathways. Unfortunately, despite extensive English literature searches, we could not find any study on the use of sulfasalazine for the treatment of sepsis and associated ARDS. Therefore, this research is the first and only study with this focus.

## Material and Methods

### Animals

Forty adult male Wistar albino rats weighing 200-250 g were used in this experiment. The rats were obtained from the Demiroğlu Bilim University Animal Experimental Laboratory, had free access to food and tap water, and were housed in temperature-controlled environments (22±2°C) in 12-h light/dark cycles.

This study was conducted in accordance with the guidelines for the care and use of laboratory animals adopted by the National Institutes of Health (USA). In addition, the experimental protocol was approved by the Animal Ethics Committee (Demiroğlu Bilim University) (number 15211108).

### Experimental procedures

The rats were randomly divided into four equal groups, and 30 were subjected to the fecal intraperitoneal injection (FIP) procedure to establish a sepsis model of intra-abdominal origin. Ten rats were used as the normal group with no procedure or treatment. The experimental model of sepsis from FIP was previously described by Sever et al. (17). In order to prepare a fecal-saline solution, the feces of rats were collected and suspended in saline at a concentration of 75 mg/mL. It was then injected intraperitoneally at a dose of 0.1 mg/kg in a single application. Study groups were constructed as follows: Group 1: Normal control (n=10) [non-surgical], Group 2: FIP (n=10) [untreated septic group], Group 3: FIP+saline (placebo) (n=10) [10 mL/kg 0.9% saline (mean SD= 3.18±0.12 mL) administered intraperitoneally in a single administration], Group 4 (n=10): FIP+sulfasalazine [250 mg/kg per day sulfasalazine (Salofalk 250 mg Ali Raif, Turkey) was administered intraperitoneally in a single administration]. One hour elapsed between the FIP procedure and the start of treatments. Thoracic CT imaging was performed on all rats in the study using ketamine anesthesia 20 h after the FIP procedure. The study was completed 24 h after the FIP procedure. Eight rats died within the first 24 h (4 from the FIP, three from the FIP + 0.9% NaCl saline group, and one from the sulfasalazine group) and were excluded from the study. At the end of the study, all animals were sacrificed (cervical dislocation) with ketamine (100 mg/kg, Ketasol, Richterpharma AG, Austria)/xylazine (50 mg/kg, Rompun, Bayer, Germany) anesthesia. Blood was collected by cardiac puncture for biochemical analysis, and lung tissue was removed for histological evaluation.

### Determination of TNF-α, IL-6, IL 1-β, ANG-2, and Nrf2 levels in plasma

Plasma, IL-6, TNF-α, IL 1-β, ANG-2, and Nrf2 levels were measured using Enzyme Linked Immunosorbent Assay (ELISA) kits (Biosciences, Abcam, UK). The measurements were carried out according to the manufacturer's instructions.

### Measurement of lipid peroxidation

The presence and amount of lipid peroxidation in plasma samples were determined by measuring malondialdehyde (MDA) levels using a thiobarbituric acid reagent (TBARS). Trichloroacetic acid and TBARS reagent were added to the plasma samples, then mixed and incubated at 100°C for 60 min. After cooling to room temperature, the samples were centrifuged at 0.704 *g* for 20 min, and the absorbance of the supernatant was read at 535 nm. Values are reported as nM/mg protein.

### Histopathological examination of the lungs

Lung tissue removed for histological examination was perfused with 200 mL of 4% formaldehyde in 0.1 M phosphate-buffered saline (PBS). Randomly selected dissected lung tissues were embedded in paraffin prior to histological examination. Twenty-five separate 5-μm sections were taken from 5 blocks obtained from each lung tissue and stained with hematoxylin and eosin (H&E). All sections were photographed with an Olympus C-5050 digital camera mounted on an Olympus BX51 microscope (Japan). Two independent pathologists blinded to the study groups performed histopathological evaluations of the lungs. The main histopathological lung injury score was calculated as previously described by Sever et al. (17) Briefly, histopathologic lung injury was assessed by assessing alveolar congestion (AC), hemorrhage (H), leukocyte infiltration or aggregation in air spaces/vessel walls (AL), perivascular/interstitial edema (PE), and thickness of alveolar wall/hyaline membrane formation (TA). The severity of each item was rated between 1 (0-25%), 2 (25-50%), 3 (50-75%), and 4 (75-100%).

### CT examination of the lungs

The CT procedure was performed as previously described by Sever et al. (17). Lung CT examinations were performed with a 16-slice multi-detector sequential CT scanner (Somatom Go Now, Siemens Healthcare, Germany) in the supine position after an anesthetic injection, without contrast. Rats were deeply anesthetized with ketamine (80 mg/kg, Ketasol, Richterpharma AG)/xylazine (10 mg/kg, Rompun, Bayer), *ip*. All animals were fixed to a scanning table with appropriate equipment to avoid motion artifacts. The study scan parameters were 120 kV, variable mAs according to the automatic exposure control system, and 1-mm slice thickness. The scan area was determined to include the apex and base of the lung. The C3 vertebra, which marks the lower level of the diaphragm, was determined as the lowest point. After image acquisition, all images were reconstructed in 1-mm non-overlapping slices with a matrix size of 512×512 and a sharp reconstruction kernel (KernelBr64). All images were evaluated by three radiologists who were blinded to the study. Six areas of interest (ROI) of equal sizes (2,153 mm^2^) (2 ROIs in the upper zone, 2 ROIs in the middle zone, and 2 ROIs in the lower zone of both lungs) were placed based on the axial image parenchymal windows near the heart apex. When placing the ROI, care was taken to avoid large vessels, airways, and bone.

### Arterial blood gas analysis

Blood samples (0.2 mL) from the carotid artery of the rats in each group were collected 24 h after the operation and PaO_2_ and PaCO_2_ were analyzed using a blood gas analyzer.

### Statistical analysis

Data are reported as means±SE. All variables were considered normally distributed by the Shapiro-Wilk test normality test. Data analysis was performed using SPSS version 15.0 for Windows (IBM, USA). Student's *t*-test and one-way analysis of variance (ANOVA) with *post hoc* Bonferroni correction were used to assess parametric variables. Survival rates were analyzed using Kaplan-Meier and Cox regression tests. P-values of 0.05 or less were considered statistically significant.

## Results

### Biochemical findings

Plasma levels of inflammatory markers are summarized in Table 1. Statistically significant elevations in IL-6, IL1-β, TNF-α, and ANG-2 levels were found in the FIP sepsis induction group compared to the normal group. Plasma levels of these parameters in the FIP and FIP+saline groups were significantly higher than the control group. On the other hand, these values were significantly lower in the sulfasalazine-treated group than in the FIP+saline group.

**Table 1:** Plasma biomarker levels of the study groups.

	Normal control (n=10)	FIP ( n=6)	FIP+saline (n=7)	FIP+sulfasalazine (n=9)
MDA (nM/mg protein)	9.7±0.6	20.1±0.9*	22.5±1.2*	12.4±1.9^#^
IL-6 (pg/mL)	5.1±0.8	22654.7±2956.2**	28272.1±2337.3**	10545.3±844.9^##^
IL 1-β (pg/mL)	2.3±0.1	2178.1±148.3**	1936.2±148.7**	942.5±45.9^##^
TNF-α (pg/mL)	11.9±4.6	417.8±20.9**	445.8±32.2**	193.3±18.1^##^
ANG-2 (pg/mL)	1.83±0.1	12.5±2.2**	10.9±1.3**	4.7±0.9^##^
Nrf2 (pg/mL)	0.63±0.1	0.11±0.08*	0.14±0.06*	0.42±0.09^#^

Data are reported as means±SE. *P<0.05, **P<0.001 *vs* normal group; ^#^P<0.01, ^##^P<0.001 *vs* FIP and FIP+saline groups (one-way ANOVA with *post hoc* Bonferroni test). FIP: fecal intraperitoneal injection procedure; MDA: malondialdehyde, IL: interleukin; TNF: tumor necrosis factor; ANG: angiopoietin; Nrf2: nuclear factor-2 erythroid related factor-2.

Nrf2 levels were significantly lower in the FIP and FIP+saline groups compared to the normal group. However, in the sulfasalazine-treated group, Nrf2 levels were significantly higher than in the FIP and FIP+saline groups.

MDA levels were higher in the FIP and FIP+saline groups than in the normal group and significantly lower in the sulfasalazine treatment group (P<0.01) compared to the FIP and FIP+saline groups.

### Histopathological score and CT findings

**Figure 1:**
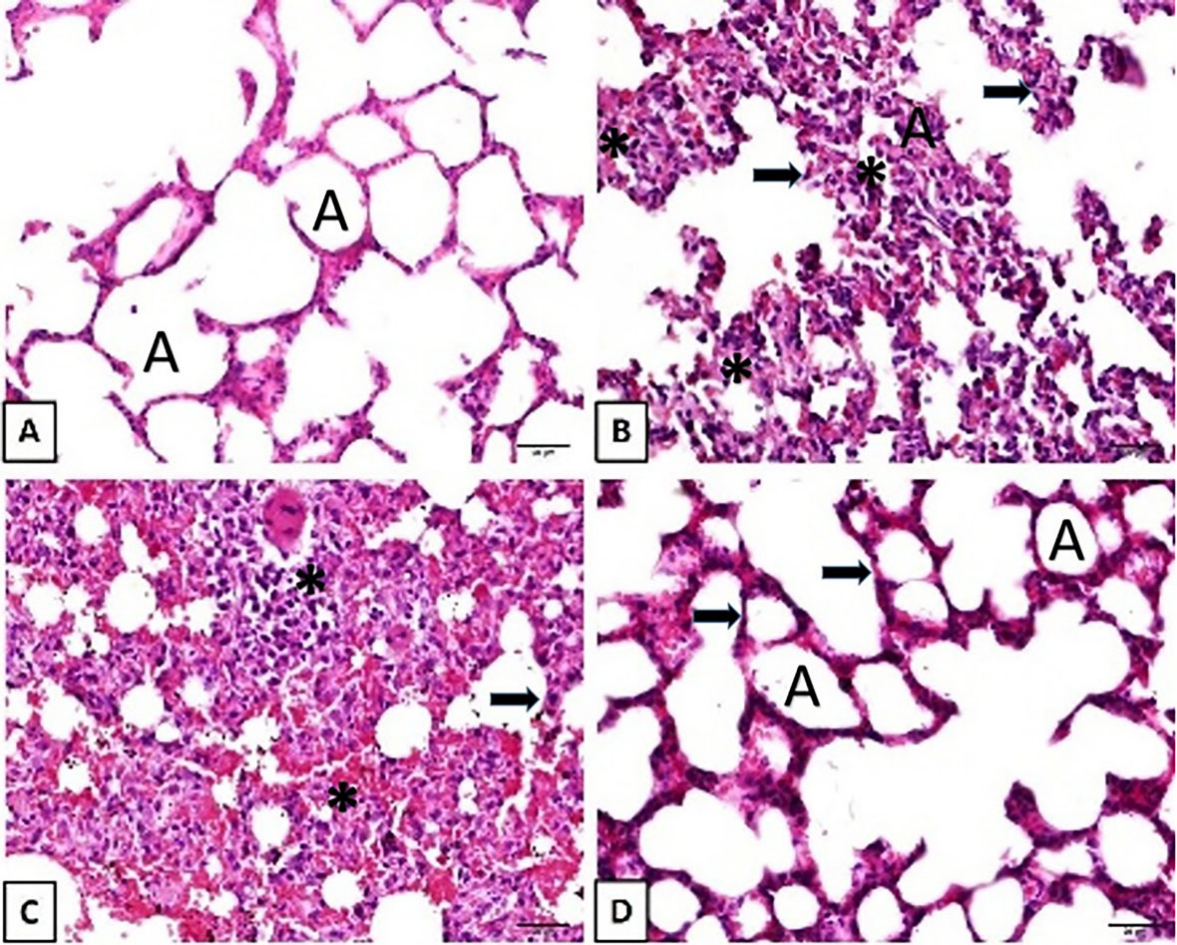
Lung histopathology with hematoxylin and eosin staining with x40 magnification (scale bar 20 μm). **A**, Normal control group lung (A: alveolus); **B**, Fecal intraperitoneal injection (FIP) group with severe histopathological alteration related to increased alveolar inflammation (*) and septal thickness (arrows); **C**, FIP and 10 mL/kg 0.9% NaCl saline group with severe histopathological alteration related to increased alveolar inflammation (*) and septal thickness (arrows); **D**, FIP and 250 mg/kg sulfasalazine group with decreased inflammation and septal thickening (arrows).

Figure 1 shows histopathological specimens of all groups. The effect of sulfasalazine in preventing the development of lung damage due to sepsis can be seen in Figure 1D. The severe inflammation seen in Figure 1B and C after sepsis induction is absent in Figure 1D, which closely resembles the normal appearance seen in Figure 1A after sulfasalazine treatment. As summarized in Table 2, the histopathological scores for alveolar congestion, hemorrhage, infiltration or aggregation of leukocytes in air spaces and vessel walls, perivascular/interstitial edema, and the thickness of alveolar wall/hyaline membrane formation in the FIP and FIP+saline groups were increased compared to the normal group. All these values, which were high and indicative of lung damage, were significantly lower in the sulfasalazine-treated group compared to the FIP+saline group.

**Table 2:** Histopathological scores and Hounsfield unit (HU) values.

	Normal control (n=10)	FIP (n=6)	FIP+saline (n=7)	FIP+sulfasalazine (n=9)
AC	0.2±0.1	3.4±0.2**	3.4±0.2**	1.1±0.2^##^
H	0.4±0.1	1.6±0.2*	1.8±0.3**	0.8±0.3^##^
AL	0.3±0.2	2.2±0.3**	2.5±0.2**	1.1±0.3^##^
PE	0.1±0.1	3.5±0.3**	3.2±0.4**	0.9±0.1^##^
TA	0.2±0.1	2.5±0.1**	2.6±0.2**	1.2±0.3^#^
CT Hounsfield unit (HU)	-640.1±8.5	-518.9±7.3**	-495.8±10.9**	-618.9±5.8^##^

Data are reported as means±SE. *P<0.01, **P<0.001 *vs* normal group; ^#^P<0.05, ^##^P<0.001 *vs* FIP+saline group (one-way ANOVA with *post hoc* Bonferroni test). FIP: fecal intraperitoneal injection procedure; AC: alveolar congestion; H: hemorrhage; AL: infiltration or aggregation of leukocytes in air spaces and vessel walls; PE: perivascular/interstitial edema; TA: thickness of alveolar wall/hyaline membrane formation.

**Figure 2:**
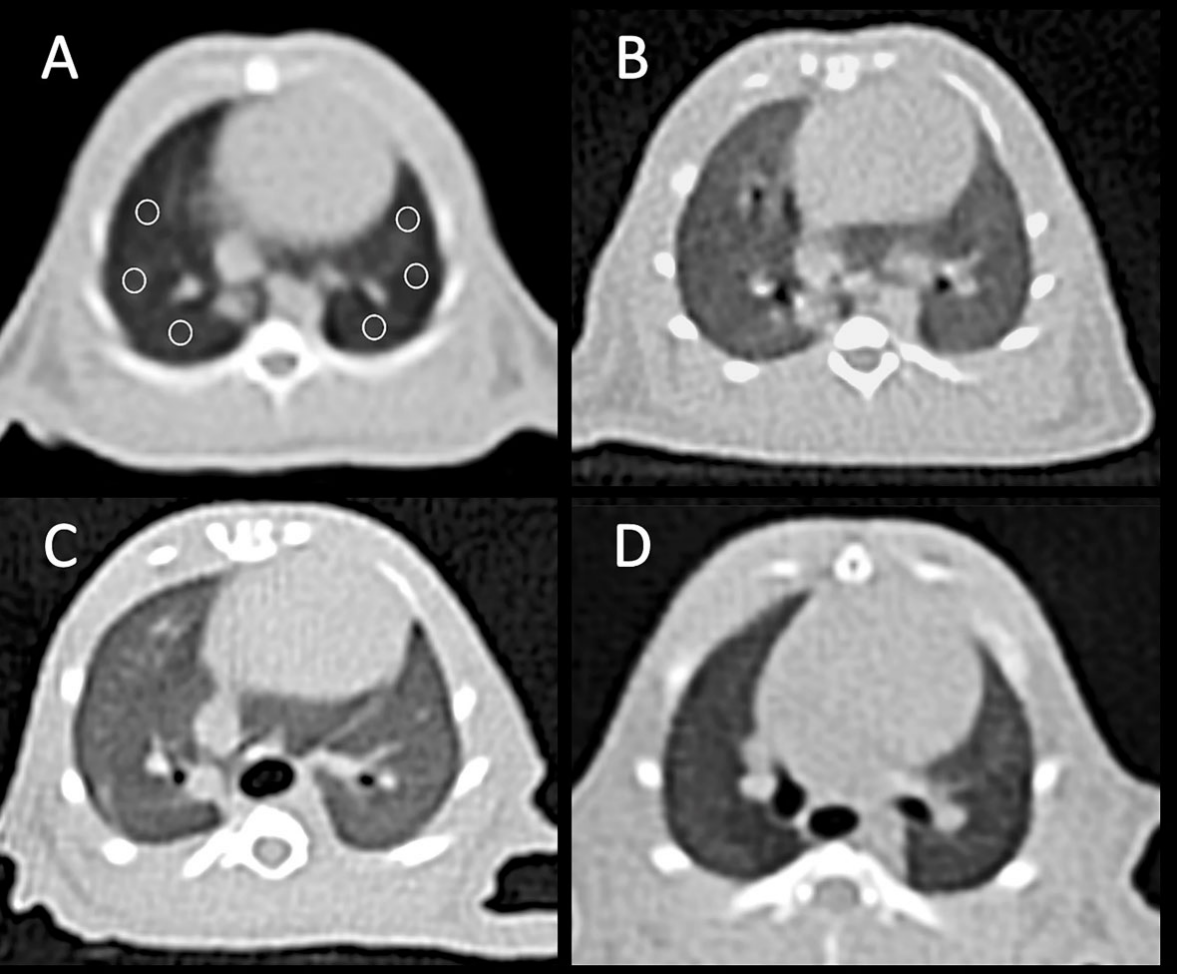
Axial non-contrast computed tomography (CT) images of the lung at the level of the heart with six regions of interest (ROI) placed with the same size and location in each group. **A**, Normal Control group; **B**, Fecal intraperitoneal injection (FIP) group showing increased density of lung; **C**, FIP and saline (placebo) group showing increased density of lung; **D**, FIP and sulfasalazine group showing a lung with density similar to the normal group.

HU units were significantly increased in the FIP (P<0.001) and FIP+saline (P<0.001) groups compared to the normal group. However, this value was significantly lower in the sulfasalazine group compared to the FIP+saline group (P<0.001). After treatment with sulfasalazine, the lung tissue density (-618.9±5.8) was found to be almost similar to the normal HU values of all groups, as shown in Table 2. The areas where HU values were measured in the lungs are shown in Figure 2.

### Arterial PaO_2_ and PaCO_2_ levels

PaO_2_ and PaCO_2_ levels in the FIP and FIP+saline groups were significantly lower compared to the normal group (all P<0.05). PaO_2_ values were significantly higher in the sulfasalazine-treated group than in the FIP+saline group (P<0.05). There was no statistically significant difference in PaCO_2_ values in these two groups. Arterial gas partial pressures are summarized in Table 3.

**Table 3:** Arterial blood gas values in study groups.

	Normal control (n=10)	FIP (n=6)	FIP+saline (n=7)	FIP+sulfasalazine (n=9)
PaO_2_ (mmHg)	103.7±9.9	79.1±7.2*	81.3±8.5*	92.1±6.6^#^
PaCO_2_ (mmHg)	40.1±4.6	32.5±8.1*	33.4±5.5*	30.9±7.1

Data are reported as means±SE. *P<0.05 *vs* normal group; ^#^P<0.05 *vs* FIP+saline group (one-way ANOVA with *post hoc* Bonferroni test). FIP: fecal intraperitoneal injection procedure; PaO_2_: partial pressure of oxygen; PaCO_2_: partial pressure of carbon dioxide.

### Survival analyses of groups

**Figure 3:**
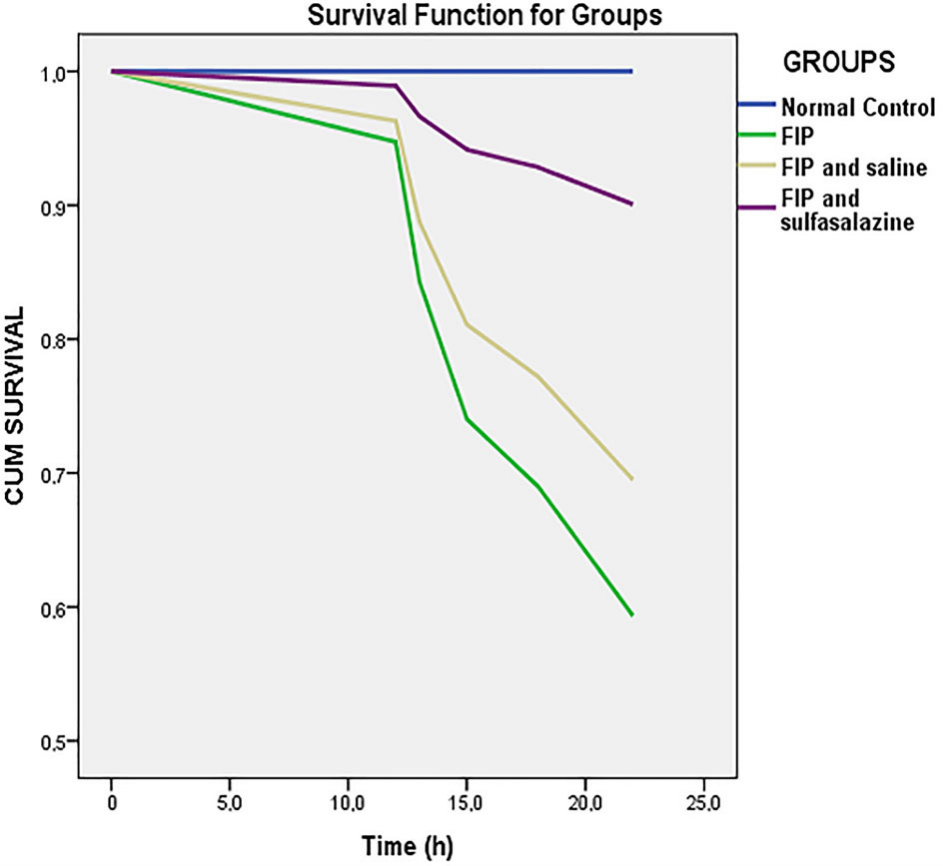
Kaplan-Meier survival curves of the four groups. In the visual evaluation, survival in the fecal intraperitoneal injection (FIP) and sulfasalazine group seems to be better than in the other FIP groups. The curve comparison with Cox regression test revealed statistically significant differences (P<0.05).

Visual examination of Kaplan-Meier curves clearly shows that there was a survival advantage in the group treated with sulfasalazine. The better survival was confirmed in the Cox regression test (P<0.05). Survival analyses of all groups are shown in Figure 3.

## Discussion

In our study, ARDS was successfully produced by the FIP application-induced sepsis, as evidenced by impaired arterial blood gasses, increased pro-inflammatory cytokines and oxidative stress markers, decreased Nrf2 levels, and pulmonary computed tomography and histopathological findings. Through this research, sulfasalazine was shown to be effective in preventing and reversing ARDS. Compared to the FIP+saline (placebo) group, sulfasalazine had a protective and curative effect against ARDS.

ARDS is characterized by an excessive inflammatory response, oxidative stress, and vascular and endothelial permeability (16). Acute lung damage is triggered by a misdirected inflammatory response. Microbial products and endogenous molecules from cell injury bind to Toll-like receptors on the lung epithelium and alveolar macrophages and induce the innate immune system (18). Although the innate immune system is very successful at trapping pathogens through methods such as extracellular neutrophil trapping and histone release, it has side effects that may increase alveolar damage (19). The immune system also produces pathogen neutralizing agents such as leukocyte proteases, reactive oxygen species, cytokines, and chemokines. Unfortunately, these also worsen alveolar damage (20). Due to the increased inflammatory response, pro-inflammatory cytokines are excessively increased. These are, in particular, TNF-α, IL-1β, IL-6, and IL-8. Our study found statistically significant increases in IL-6, IL1-β, and TNF-α levels in the FIP groups compared to the normal group. Thus, there were signs of an increased inflammatory response. However, statistically significant decreases were noted in the sulfasalazine treatment group compared to the placebo group. Correcting a compromised immune system is the most critical treatment approach, and sulfasalazine has been successfully used to inhibit increased immune responses.

Sulfasalazine may exert this effect by activating the Nrf2 pathway of oxidative stress. Our study observed statistically significant decreases in serum Nrf2 levels in the sepsis groups and a significant increase in the sulfasalazine-treated group compared to the placebo group. Sepsis initiates the development of ARDS by inducing oxidative stress. Oxidative stress is a common condition defined as an imbalance between ROS and antioxidant capacity in cells under transient or constant stimulation by oxidative stressors. In response to an excitatory event such as lipopolysaccharide (LPS) production due to bacterial infection, the lung macrophages and endothelium are activated and upregulate the surface expression of adhesion molecules. Neutrophils are an important source of ROS (21). Under normal conditions, Nrf2 binds to its repressor Keap1 and undergoes proteasome degradation after being tagged by ubiquitination. However, under conditions of oxidative stress, Nrf2 is translocated to the nucleus, where it dimerizes with small members of the Maf family, binds to ARE genes, and increases enzymes such as heme oxygenase (HO-1). Elevated HO-1 catalyzes heme to carbon monoxide (CO), bilirubin, and free iron. CO acts as an inhibitor of nuclear factor-kB (NF-kB) signaling. This inhibits the release of pro-inflammatory cytokines. The other product, bilirubin, acts as an antioxidant.

In addition, HO-1 directly inhibits pro-inflammatory cytokines and activates anti-inflammatory cytokines, thereby balancing the inflammatory process (11). As a result of the increase in Nrf2 levels by sulfasalazine, a decrease in NF-κB levels occurred in our study and, accordingly, a decrease in pro-inflammatory cytokines and an increase in the levels of antioxidant levels also occurred. Sulfasalazine is a potent NF-κB inhibitor and anti-inflammatory agent (22). Apart from the known inhibitory effect of sulfasalazine on NF-κB, it also inhibits it via the Nrf2 pathway. Therefore, sulfasalazine may increase Nrf2 levels in the cell, further decreasing NF-κB and pro-inflammatory cytokines. This effect is caused by a crosstalk between Nrf2 and NF-κB. A study by Kim et al. (23) showed that sulfasalazine increased HO-1 enzyme by increasing Nrf2 levels. Hyperoxia or LPS-induced ARDS are perhaps two of the best-studied models that benefit from Nrf2 activation, and Nrf2-deficient mice are used extensively in studies focusing on the beneficial role of Nrf2 in ARDS (24). Furthermore, Nrf2-deficient mice are more likely to develop ARDS with increased lung hyperpermeability, epithelial injury, and inflammation under stimulation of hyperoxia and butylated hydroxytoluene compared to wild-type mice (25). These effects occur through the reduction of oxidative stress.

As a result of lipid peroxidation after oxidative stress, the end product MDA is formed. It is a reliable marker for lipid peroxidation, a well-established mechanism of cell damage, and is used as an indicator of oxidative stress (26). Therefore, MDA levels were used in our study as an indicator of oxidative stress. MDA levels were significantly higher in the sepsis-induced groups compared to the normal control group. However, in the sulfasalazine-treated group, MDA levels were significantly lower than in the sepsis + saline (placebo) group (Table 1). This result tells us that sulfasalazine reduced oxidative stress.

ANG-2 is a growth factor that is part of the angiopoietin/Tie signaling pathway, one of the major pathways involved in angiogenesis (27). Under normal physiological conditions, ANG-2 levels are relatively low, but their levels are elevated in aggressive conditions such as inflammation or cancer. By acting on endothelial cells, ANG-2 increases endothelial permeability and causes vascular leakage, infiltration of immune cells into the endothelium, and further worsening of the condition. Therefore, ANG-2 has been proposed as a marker for inflammatory diseases and cancer (28). Like ANG-1, ANG-2 binds to the Tie2 receptor with the same binding affinity, inducing its antagonistic role, but does not bind to Tie1 (29). ANG-2 expression is triggered by inflammatory mediators such as thrombin (30) and conditions such as hypoxia (31) and cancer (32). ARDS is a life-threatening condition characterized by loss of endothelial cell barrier function, which can lead to hemorrhagic shock and sepsis. In the study by Lomas-Neira et al. (33), high ANG-2 levels were consistent with poor prognosis of the disease, and decreased ANG-2 prevented hemorrhagic shock and septic events. Plasma ANG-2 has been reported as a potential causative marker for sepsis-associated ARDS (34). Our study found a statistically significant ANG-2 elevation in the sepsis groups compared to the normal control group.

On the other hand, a significant lower ANG-2 serum level was found in the sulfasalazine-treated group compared to the sepsis + saline (placebo) group. With this effect, sulfasalazine significantly reduced fluid and inflammatory cell levels in the alveoli by reducing vascular leakage. Studies show that the higher the ANG-2 score, the lower the likelihood of recovery from ARDS and sepsis (35). Sulfasalazine probably achieves this reducing effect through its anti-inflammatory effects. TNF-α triggers the formation of ANG-2 (36). Sulfasalazine reduces TNF-α production and, accordingly, ANG-2 production. A study showing the relationship between ANG-2 and sulfasalazine could not be found in the literature. We predicted this in the hypothesis we made at the beginning of the study. Our prediction was consistent with the study results.

The effect of sulfasalazine in preventing ARDS was also assessed using the CT imaging technique (Figure 2). In our study, the HU values in the sepsis groups were higher than in the normal group. However, statistically significant decreases in HU scores were noted in the sulfasalazine-treated group compared to the sepsis + saline (placebo) group (Table 2). CT is the gold standard imaging technique for assessing lung morphology and performing quantitative analysis of lung tissue ventilation and recruitment (37). CT scans allowed us to easily see the non-ventilated and infiltrated areas and provided numerical values (7). We also demonstrated the protection achieved with sulfasalazine observed by histopathological evaluation by converting the histological scores into numerical values. We found that these values were significantly higher in the sepsis groups compared to the normal control group. However, after sulfasalazine treatment, these values decreased significantly compared to the placebo group (Table 2). These results showed that sulfasalazine protected and healed lung tissue.

There was a statistically significant increase in PaO_2_ levels in the sulfasalazine-treated group compared to the placebo group. There were 8 deaths in the study, including 4 in the FIP, 3 in the FIP+saline, and one in the FIP+sulfasalazine group. The majority of deaths occurred in the untreated FIP and FIP+saline groups. The Kaplan-Meier probability curves and Cox regression test (P<0.05) showed greater survival for the FIP+sulfasalazine group. Although the cause of death of the rats is thought to be sepsis, the PaCO_2_ values suggested that they may be low due to the compensation of metabolic acidosis.

In sepsis, lactic acid-induced metabolic acidosis occurs and PaCO_2_ levels decrease to compensate. There was a decrease in PaCO_2_ values in all sepsis groups compared to the normal group. This indicates metabolic acidosis in the sepsis groups and a decrease in CO_2_ levels to compensate for this. This shows that acidosis continued in the group treated with sulfasalazine and that sulfasalazine treatment did not have a positive effect on acidosis. It is very likely that the cause of death of the rats was acidosis. The continuation of acidosis in living rats also supports this negative situation. However, the group treated with sulfasalazine had better survival, which may be due to the effect of sulfasalazine on innate immunity and antioxidant mechanisms.

This study had some limitations. One of them is the short duration of the study. The entire study was completed in 24 h. Longer survival follow-ups would be better to evaluate the protective and possible therapeutic effect of sulfasalazine. For this reason, similar studies should be performed with intra-abdominal sepsis models and longer follow-up, in which the cecum is ligated and perforated.

The second limitation is that respiratory rate and pH values were not assessed in the study, which could provide definitive evidence for our evaluation of acidosis. These values can be added in a similar study in the future.

In conclusion, this study demonstrated that sulfasalazine may be effective in preventing ARDS due to intra-abdominal sepsis as demonstrated by radiological, histological, and biochemical findings. In addition, survival curves support the protective properties of sulfasalazine in ARDS originating from intra-abdominal sepsis. Furthermore, we showed that sulfasalazine can achieve this through its effect on Nrf2 and ANG-2 signaling pathways, as hypothesized. Undoubtedly, our findings must be supported by further studies, but in the future, sulfasalazine, an old drug, may be involved in the treatment of ARDS, an unsolved medical problem.
